# Regulation of Na^+^ and K^+^ homeostasis in plants: towards improved salt stress tolerance in crop plants

**DOI:** 10.1590/1678-4685-GMB-2016-0106

**Published:** 2017-03-27

**Authors:** Diego M. Almeida, M. Margarida Oliveira, Nelson J. M. Saibo

**Affiliations:** 1 Genomics of Plant Stress Unit, Instituto de Tecnologia Química e Biológica António Xavier, Universidade Nova de Lisboa and Instituto de Biologia Experimental e Tecnológica, Oeiras, Portugal.

**Keywords:** Salinity, sodium, potassium, proton pumps, ion transporters

## Abstract

Soil salinity is a major abiotic stress that results in considerable crop yield losses worldwide. However, some plant genotypes show a high tolerance to soil salinity, as they manage to maintain a high K^+^/Na^+^ ratio in the cytosol, in contrast to salt stress susceptible genotypes. Although, different plant genotypes show different salt tolerance mechanisms, they all rely on the regulation and function of K^+^ and Na^+^ transporters and H^+^ pumps, which generate the driving force for K^+^ and Na^+^ transport. In this review we will introduce salt stress responses in plants and summarize the current knowledge about the most important ion transporters that facilitate intra- and intercellular K^+^ and Na^+^ homeostasis in these organisms. We will describe and discuss the regulation and function of the H^+^-ATPases, H^+^-PPases, SOS1, HKTs, and NHXs, including the specific tissues where they work and their response to salt stress.

## Salt stress effects on plant growth and yield

Soil salinity is a major environmental constrain to crop production, affecting millions of hectares of land throughout the world and costing billions of dollars every year (Munns, 2005; Munns and Tester, 2008; Shabala and Cuin, 2008). High salinity affects over 6% of the world's total land area. Most of this affected land has arisen from natural causes, such as rainfall, windblown salt from ocean, tsunamis, and rock weathering. Apart from natural causes, soil salinization is commonly associated to land clearing by removal of deep root vegetation, thus accumulating more water and consequently raising the levels of salty groundwater, or irrigation practices, such as the use of water with high salt concentration. Currently it is estimated that 20% of the total irrigated land is salt-affected. Given that irrigated land produces at least twice as much as rain-fed land and is responsible for one third of the world's food production, it raises awareness for salinity as a serious problem for crop productivity (Munns, 2005; Munns and Tester, 2008).

High soil salinity is a condition characterized by a high concentration of soluble salts, in which NaCl is the most soluble and widespread salt. Soils are classified as saline when the electrical conductivity (EC) is 4 dS/m (≈ 40 mM NaCl) or higher. At this soil salt concentration, growth and yield of most crops are significantly reduced. Rice, as well as most crop plants, is a glycophyte and therefore it can only tolerate relatively low concentrations of salt. Among cereal crops, rice is the most salt sensitive one, showing salt stress symptoms and reduced yield even when the EC is lower than 4.0 dS/m (Munns and Tester, 2008). The salinity threshold for rice is 3.0 dS/m with a 12% reduction in yield per dS/m beyond this threshold (Gao *et al.*, 2007). However, some degree of genotype diversity for salt stress tolerance is available in rice germplasm. Among 180,000 rice genotypes screened by the International Rice Research Institute (IRRI, 2013), 17% showed acceptable tolerance at an EC of 10 dS/m at seedling stage (Gregorio *et al.*, 2002).

High salinity affects plants in two distinct phases. The first phase is the osmotic effect, which is independent of the accumulation of salt in the shoot. Salts dissolved in the soil solution reduce the soil water potential. This makes the water uptake from roots thermodynamically hampered and induces water deficit (Pardo, 2010; Roy *et al.*, 2014). A water deficit signal is rapidly transmitted (within minutes) from roots to shoots and will cause intracellular turgor reduction and decreased cell expansion (Munns, 2005; Munns and Tester, 2008). This signal also promotes the biosynthesis of abscisic acid (ABA), which leads to a lower stomatal conductance (Munns, 2005; Munns and Tester, 2008; Roy *et al.*, 2014). The lower stomatal conductance causes a lower carbon assimilation, biomass production and decreased yield. The second phase of salinity is ionic specific; this is due to the accumulation to toxic concentrations of sodium (Na^+^) and/or chloride (Cl^−^) ions, especially in the older leaves, inducing tissue necrosis and early leaf senescence (Roy *et al.*, 2014). For most plant species Na^+^ appears to reach a toxic concentration earlier than Cl^−^ (Tester and Davenport, 2003). For rice Na^+^ has been shown to be the primary toxic ion (Chi Lin and Huei Kao, 2001; Tsai *et al.*, 2004). Both osmotic and ionic effects disturb aerobic metabolism and induce the accumulation of reactive oxygen species (ROS) beyond the plant's capacity for cellular oxidant detoxification, which in turn negatively affects cellular structures and metabolism (Chaves and Oliveira, 2004; Chaves *et al.*, 2009).

A deleterious effect imposed by salt stress, during the second phase, is ion imbalance (Munns and Tester, 2008). Potassium (K^+^) is an essential macronutrient that plays important functions related to enzyme activation, osmotic adjustment and turgor generation, regulation of membrane potential, and cytoplasmatic pH homeostasis (PPI, 1998; Barragan *et al.*, 2012). Due to similarity in physicochemical properties between Na^+^ and K^+^ (*i.e.*, ionic radius and ion hydration energy), the former competes with K^+^ for major binding sites in key metabolic processes in the cytoplasm, such as enzymatic reactions, protein synthesis and ribosome functions (Marschner, 1995; PPI, 1998). Na^+^ inhibits the enzyme activity of many enzymes that require K^+^ for functioning (Duggleby and Dennis, 1973). With over 50 different cytoplasmic enzymes being activated by K^+^, disruption of the K^+^ homeostasis leads to severe metabolism impairment, both in root and leaf tissues (Marschner, 1995; PPI, 1998). It has been suggested that plant survival under salt stress requires a high cytosolic K^+^/Na^+^ ratio in the cytoplasm. The restriction of Na^+^ accumulation in shoots under salt stress has been correlated with salt stress tolerance in rice (Lutts *et al.*, 1996) and maize (*Zea mays* L.) (Tester and Davenport, 2003).

## Sodium uptake from soil, sensing and signaling mechanisms

**Figure 1:**
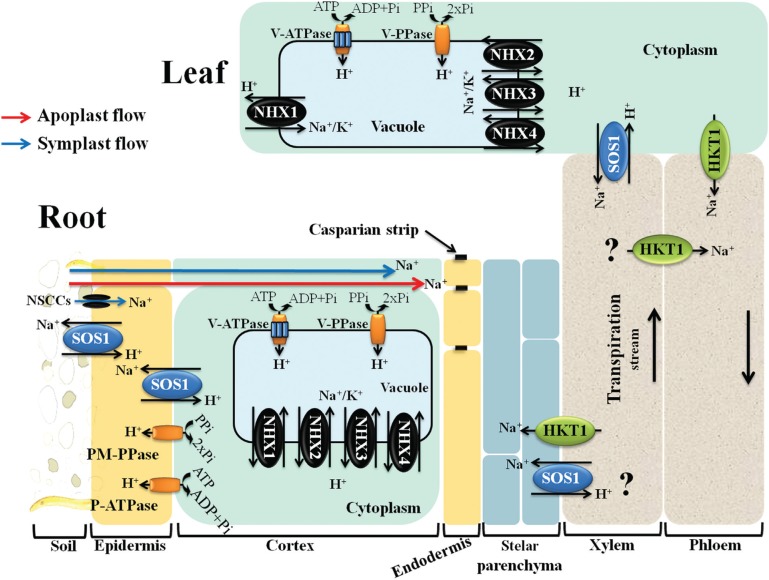
Schematic representation showing key plasma and tonoplast membrane transporters, channels and pumps mediating Na^+^ and K^+^ homeostasis in plants under salt stress (adapted from Roy *et al.*, 2014). Na^+^ ions enter the cells via Non Selective Cation Channels (NSCCs) and possibly via other cation transporters not shown (symplast flow - blue arrow) and through the cell wall and intercellular spaces (apoplast flow - red arrow). The Na^+^/H^+^ antiporter SOS1 extrudes Na^+^ at the root soil interface, thus reducing the Na^+^ net influx of Na^+^. At the xylem parenchyma cells, HKT1-like proteins retrieve Na^+^ from the xylem sap, thereby restricting the amount of Na^+^ reaching the photosynthetic tissues. To translocate Na^+^ back to the root, ions unloaded from xylem may be transported into phloem via additional HKT1-like protein. In addition, HKT1-like proteins also load Na^+^ into shoot phloem, and then Na^+^ is transferred into roots via phloem, preventing Na^+^ accumulation in shoots. SOS1, localized in the xylem parenchyma cells, is also suggested to mediate Na^+^ efflux from xylem vessels under high salinity. Incoming Na^+^, in root and shoots, is stored in the large central vacuole by tonoplast-localized NHX exchangers (NHX1-4). Plasma membrane (PM) H^+^-ATPase (P-ATPase), PM H^+^-PPase (PM-PPase), tonoplast H^+^-ATPase (V-ATPase) and tonoplast H^+^-PPase (V-PPase) generate electrochemical potential gradient for secondary active transport.

The very low membrane potential across the plasma membrane of root cells (more negative inside) promotes the passive transport of Na^+^ into the cells, and especially so when the sodium concentration increases in the soil solution. In contrast, Na^+^ efflux (*i.e.*, removal from the cell) is not passive and requires energy expenditure (Maathuis *et al.*, 2014). The passive Na^+^ uptake into root cells at high soil salinity is mainly mediated by a family of Non-Selective Cation Channels (NSCCs family), for which the molecular identity remains largely unknown (Blumwald *et al.*, 2000; Kronzucker and Britto, 2011) (Figure 1). In addition to the Na^+^ flow across cellular membranes to enter into root cells (symplast flow), it has been reported that, at least in some species, interruptions in the endodermis (passage cells) allow the movement of water and solutes (*i.e.*, Na^+^) through the cell wall and intercellular spaces. This type of transport into the xylem stream, without crossing the plasma membrane, is referred as “apoplast flow” (Yeo *et al.*, 1987; Kronzucker and Britto, 2011) (Figure 1). Casparian strips and suberine layers in the root endoderm and exodermal layers provide some barrier to apoplast flow (Yeo *et al.*, 1987). In many plant species, such as rice, the apoplast flow is considered to be the major port of Na^+^ entry (≈ 50% of total Na^+^ uptake) (Yeo *et al.*, 1987), especially at high salinity levels, and is responsible for a significant amount of Na^+^ transported to the shoot (Yeo *et al.*, 1987; Kronzucker and Britto, 2011). Na^+^ ions taken up by the roots are then transported to shoots via xylem vessels by bulk flow (Figure 1). This is driven by the tension in the xylem, which causes the continuous movement of water from the root through the plant to the surrounding atmosphere during transpiration (Nobel, 2009).

Sodium has also a strong inhibitory effect on K^+^ uptake by cells, probably by inhibiting K^+^ transporters, such as AKT1 (hyperpolarization-activated inward-rectifying K^+^ channel), a major player in K^+^ acquisition by plants (Hirsch *et al.*, 1998; Fuchs *et al.*, 2005), and HAK5 (carrier-type HUP/HAK/KT transport) (Nieves-Cordones *et al.*, 2010), both present in the plasma membrane of root cells. Additionally, membrane depolarization caused by large cytosolic Na^+^ influx results in increased K^+^ efflux, possible through depolarization-activated outward-rectifying K^+^ channels (*e.g.*, GORK) (Adams and Shin, 2014) and also NSCCs (Sun *et al.*, 2009).

**Figure 2:**
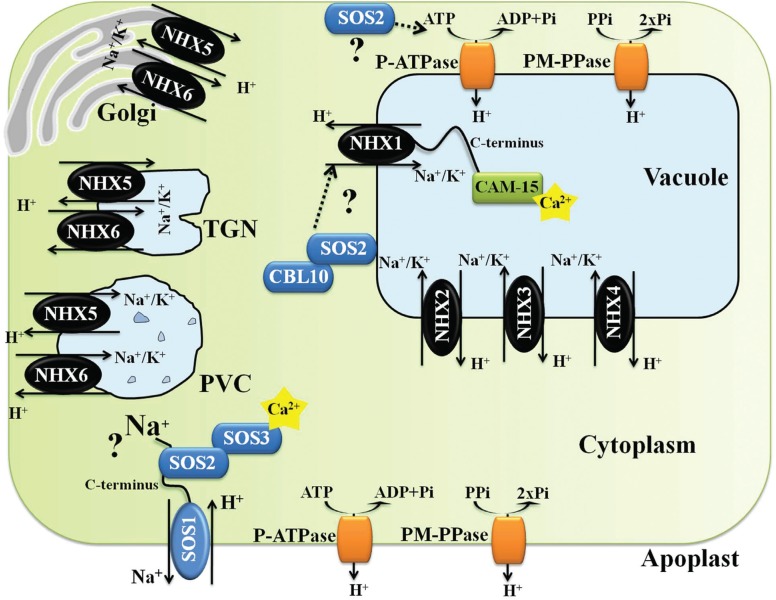
Schematic representation of a hypothetical Arabidopsis cell indicating subcellular localizations, functions, and regulations of NHXs antiporters (NHX1-6), plasma membrane H^+^-ATPase (P-ATPase), tonoplast H^+^-ATPase (V-ATPase), tonoplast H^+^-PPase (V-PPase) and SOS1 (adapted from Bassil *et al.*, 2012). *Trans*-Golgi network (TGN), and prevacuolar compartment (PVC).

Very little is known about how Na^+^ is sensed in most cellular systems. In theory, Na^+^ can be sensed either outside or inside the cell, or both. Extracellular Na^+^ may be sensed by a membrane receptor, whereas intracellular Na^+^ may be sensed either by membrane proteins or by any of the Na^+^ sensitive enzymes in the cytoplasm (Conde *et al.*, 2011). The plasma membrane Na^+^/H^+^ antiporter SOS1 (SALT OVERLY SENSITIVE 1) has been described as a possible Na^+^ sensor (Shi *et al.*, 2000). Its transport activity is essential for Na^+^ efflux from cells (Quintero *et al.*, 2002), but its unusually long cytoplasmatic tail is thought to be involved in Na^+^ sensing (Shi *et al.*, 2000) (Figure 2). However, this mechanism it is not fully clear.

In plant cells, Ca^2+^ acts as a second messenger connecting a wide range of extracellular stimuli with various intracellular responses (Conde *et al.*, 2011). Salt stress originates a fast and transient increase in free cytosolic Ca^2+^, likely released from the vacuole (Pottosin *et al.*, 2009), which is decoded by Ca^2+^ sensors, such as calmodulin (CaM), calcineurin B-like proteins (CBLs) and CBL-interacting protein kinases (CIPKs). When acting as a CBL-CIPK complex, these Ca^+^ sensors are often designed as calcium-dependent protein kinases (CDPKs) (Yang and Poovaiah, 2003; Conde *et al.*, 2011). Cytosolic Ca^2+^ sensors in turn trigger many signal transduction pathways involved in the regulation of ion channels activity (*e.g.*, NSCCs are strongly blocked by external Ca^+2^), as well as enzymatic activity and gene transcription, ending up in ion homeostasis (Pardo and Quintero, 2002; Yamaguchi *et al.*, 2005; Martínez-Atienza *et al.*, 2007; Conde *et al.*, 2011; Adams and Shin, 2014).

## Mechanisms of salt tolerance in plants

Salt stress frequently affects plant habitats and many species evolved different mechanisms to cope with it. The mechanisms for salt tolerance can be classified into three main categories. The first one is osmotic stress tolerance, which is regulated by long distance signals that reduce shoot growth (Roy *et al.*, 2014) and involves biosynthesis and accumulation of compatible solutes to maintain water uptake (Peleg *et al.*, 2011). The second mechanism is ion exclusion, in which Na^+^ transporters reduce the accumulation of toxic Na^+^ within roots and leaves. This system operates by controlling the Na^+^ loading into the xylem and Na^+^ retrieval from the xylem, before reaching the photosynthetic tissues in the shoot (Figure 1). Finally, the third mechanism is tissue tolerance, in which high salt concentration is found in leaves, but Na^+^ is compartmentalized at the cellular and intracellular level (especially in the vacuole) reducing the deleterious effect of Na^+^ in the cytosol and driving water uptake to cells (Figure 1) (Munns and Tester, 2008). In most cases, the plant salt stress tolerance relies on the three mechanisms together, rather than on only one mechanism in particular (Munns and Tester, 2008; Roy *et al.*, 2014; Pires *et al.*, 2015).

## Sodium transporters and plant salt stress tolerance

The study of salt stress tolerance in plants usually focuses on the control of Na^+^ movement, namely on: Na^+^ exclusion from roots, Na^+^ long distance transport, and Na^+^ compartmentalization at both cellular and tissue level (Munns, 2005; Conde *et al.*, 2011; Roy *et al.*, 2014). These processes are mediated by membrane transporters, reason why the manipulation of their activity has an enormous potential to improve plant performance under high salinity (Brini and Khaled, 2012). Here, we focus on the specific membrane transporters involved in the above outlined tolerance processes. In contrast to animal cells, higher plants do not have Na^+^-ATPases or K^+^/Na^+^-ATPases and, rely on H^+^-ATPases and H^+^-pyrophosphatases (PPases) to create the proton-motive force necessary to drive Na^+^ transport across membranes (Conde *et al.*, 2011). The plasma membrane localized SOS1 (Martínez-Atienza *et al.*, 2007; Ji *et al.*, 2013) and the vacuole membrane (tonoplast) localized NHX1 (Jiang *et al.*, 2010; Fukuda *et al.*, 2011) are two Na^+^/H^+^ antiporters involved in Na^+^ exclusion back to the soil and in Na^+^ compartmentalization in the vacuole, respectively. In addition, members of the HKT1 family of HKTs (High Affinity Potassium Transporters) are involved in the control of Na^+^ long distance transport by reabsorption of Na^+^ from the xylem sap into the root cells, preventing the large accumulation of Na^+^ in the above-ground tissues (Rus *et al.*, 2004) (Figure 1). It is noteworthy that the HKT1 Na^+^ exclusion mechanism from the transpiration stream has been frequently indicated as a strong trait in salt tolerance of different cereals, such as rice (Ren *et al.*, 2005) and durum wheat (*Triticum turgidum* L. subsp. *durum*) (James *et al.*, 2006).

In the following sections, the role that different Na^+^ transporters and H^+^-pumps play in plant salt stress response is discussed.

## H^+^-Pumps and the plant response to salt stress

Proton gradients are crucial for the transport of ions and solutes across the different plant cell membranes. Three primary proton transport proteins are found in plant cells: (1) plasma membrane (PM) and (2) vacuolar H^+^-ATPases, which couple ATP hydrolysis with proton transport, and (3) PM and vacuolar H^+^-PPase, which couple pyrophosphate hydrolysis with proton transport (Gaxiola *et al.*, 2007; Fuglsang *et al.*, 2010). The H^+^-Pumps generate an electrochemical potential gradient across membranes, which is the motive force for a large set of secondary transports.

### Plasma membrane H^+^-ATPase

The PM H^+^-ATPase belongs to a class known as P-type ATPases (P-ATPases), and is encoded by a large gene family (Gaxiola *et al.*, 2007; Fuglsang *et al.*, 2010). The pump is formed by a single subunit protein, which contains 10 trans-membrane helices and a large cytoplasmatic domain (Fuglsang *et al.*, 2010). Arabidopsis and rice genomes encode 11 and 10 P-ATPases, respectively (Axelsen and Palmgren, 2001; Arango *et al.*, 2003).

The proton motive force created by P-ATPases is largely responsible for a negative potential across the plasma membrane, which is essential for root nutrient uptake, stomatal aperture, phloem loading, and cell growth (Blumwald *et al.*, 2000; Gaxiola *et al.*, 2007; Mansour, 2014). Besides regulation of many physiological processes, the P-ATPases have a critical role in plant adaptation to salt stress conditions. Higher P-ATPases activity under salt stress conditions repolarizes the NaCl-induced depolarization of PM. This response has been strongly associated with salt stress tolerance (Mansour, 2014). The maintenance of the PM potential under salt stress through P-ATPases activity has a great effect on reduction of Na^+^ influx via depolarization-activated NSCCs and K^+^ efflux via KORs and NSCCs, which help to restore higher K^+^/Na^+^ levels (Sun *et al.*, 2009). The higher P-ATPases activity under salt stress also energizes the active transport that exclude Na^+^ from root cells, a process dependent of the SOS1 Na^+^/H^+^ antiporter (Gaxiola *et al.*, 2007). Furthermore, it was reported that higher activation of P-ATPases is often found in halophytes and salt tolerant genotypes, which may correlate with salt stress tolerance (Mansour, 2014). For instance, in rice *callus* lines, a higher activation of P-ATPases occurred in salt-tolerant lines as compared to less tolerant ones (Pons *et al.*, 2011).

The salt-dependent activation of PM H^+^-pump is associated with increased levels of gene expression as well as post-translational modifications of the enzyme present in a preexisting pool (Gaxiola *et al.*, 2007; Mansour, 2014). However, it is likely that regulation of the pump activity occurs mostly at post-translational level (Gaxiola *et al.*, 2007; Fuglsang *et al.*, 2010). The pump activity can be modulated by phosphorylation/dephosphorylation of the penultimate amino acid residue of the cytoplasmatic C-terminus domain, a threonine residue. The phosphorylated threonine residue promotes binding of the activating 14-3-3 protein (Fuglsang *et al.*, 2010).

Stomatal aperture involves regulation of osmotic pressure within the guard cells, a process powered by P-ATPases activity and responsive to a wide variety of external signals (Gaxiola *et al.*, 2007). Blue light perception in guard cells is mediated by phototropins, which intitiate a signal transduction signal pathway that involves an upstream protein phosphatase I and a downstream protein kinase that phosphorylates the penultimate C-terminus amino acid residue of the P-ATPase (Takemiya *et al.*, 2006; Gaxiola *et al.*, 2007). Under drought and salt stress conditions, stomatal closure is induced by ABA through a mechanism that involves production of hydrogen peroxide (H_2_O_2_) and dephosphorylation of the P-ATPases (McAinsh *et al.*, 1996; Zhang *et al.*, 2001; Gaxiola *et al.*, 2007).

### Vacuolar H^+^-ATPase

Among the three proton-pumps found in plant cells, the vacuolar H^+^-ATPase (V-ATPase) is the most complicated one (Gaxiola *et al.*, 2007). The V-ATPase was first found associated with endomembrane system where it acidifies and generates a proton force motive within diverse cell compartments (*e.g.*, vacuole, endoplasmic reticulum and *trans*-Golgi network) (Ratajczak, 2000). However, V-ATPases have also been associated with cell plasma membrane (Hanitzsch *et al.*, 2007). The ability of the V-ATPase to maintain the cytosolic pH homeostasis and to acidify the endomembrane compartments is crucial during essential processes, such as cell growth and elongation (Hanitzsch *et al.*, 2007).

**Figure 3:**
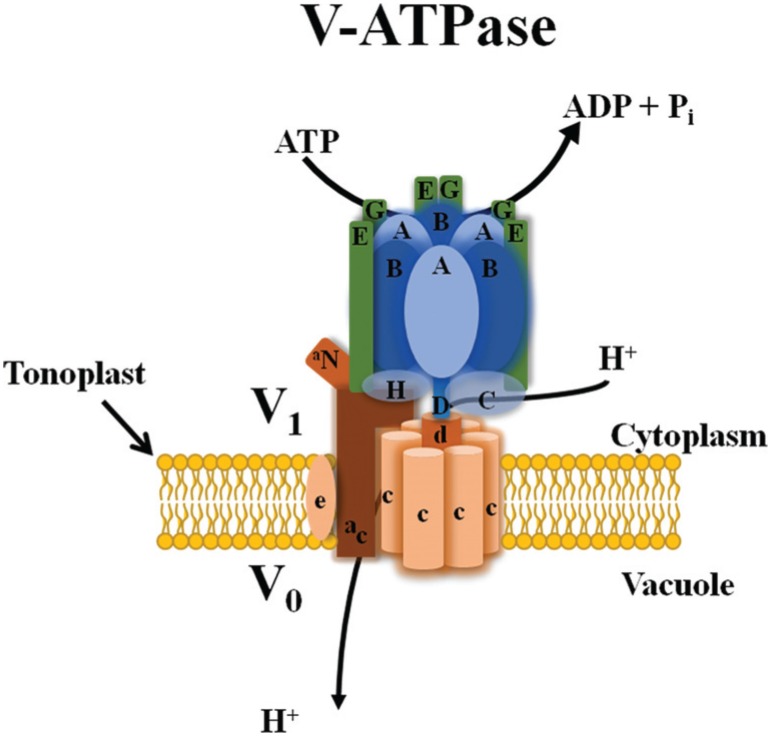
Structural model of the plant V-ATPase adapted from Gaxiola *et al.* (2007). The peripheral V_1_ complex (blue) and the membrane integral V_0_ complex (orange) are linked through a peripheral stalk formed by subunits a, C, E, G and H. Hydrolysis of ATP is coupled with H^+^ transport to the vacuole.

Vacuolar H^+^-ATPases are multisubunit enzymes composed of two subcomplexes (V_1_ and V_0_): the peripheral V_1_ complex consists of eight subunits (A, B, C, D, E, F, G and H) responsible for ATP hydrolyses, and the V_0_ membrane-integral complex consists of up to six subunits (a, c, c’, c”, d and e) responsible for proton translocation (Gaxiola *et al.*, 2007) (Figure 3). In plants, the subunit c’ is not found and many of the V-ATPase subunits are encoded by gene families. In Arabidopsis and rice, the 13 subunits which compose the vacuolar H^+^-ATPases (A, B, C, D, E, F, G, H, a, c, c”, d and e) are encoded by a total of 27 genes and 22 genes respectively (known as VHA genes). If all possible isoform combinations are used, we will have hundreds of different V-ATPase complexes (Sze *et al.*, 2002; Hanitzsch *et al.*, 2007).

By convention, the subunits of V_1_ and V_0_ complexes are distinguished with capital and case letters, respectively. The V_1_ complex consists of: (1) a globular hexameric head with three alternating copies of subunits A and B forming a ring, (2) a central rotational stalk composed of single copies of subunits D and F, and (3) a outer stalk made of subunits C, E, G and H. Subunits A and B mediate the hydrolysis of ATP at three reaction sites associated with subunit A. Both the central rotational stalk and fixed outer stalk connect the V_1_ complex to the membrane inserted V_0_ complex. The proton transporting V_0_ complex consists of six or more c subunits, also forming a ring structure. In addition, each V_0_ complex contains one copy of subunits a, d and e (Beyenbach and Wieczorek, 2006; Hanitzsch *et al.*, 2007) (Figure 3). It has been reported that structural changes of the V-ATPase complex or presence/absence of individual protein isoforms could be correlated with differences in V-ATPase localization and activity between plant organs and/or tissues (Gaxiola *et al.*, 2007).

The plant vacuole plays a very important role in the maintenance of cellular metabolism due to its role in long term storage of toxic ions, long or short term storage of mineral and/or organic acids and in pH and Ca^2+^ cytoplasmatic homeostasis. Furthermore, the V-ATPase is the most abundant H^+^-pump in the tonoplast and it has been shown that its activity is modulated to cope with environmental and metabolic changes (Ratajczak, 2000). For instance, under salt stress, a general increase of V-ATPase activity has been reported in many plant species (Matsumoto and Chung, 1988; Silva and Gerós, 2009). The V-ATPase provides the driving force necessary for Na^+^ vacuole compartmentalization, a process related on the NHX1 antiporter activity (Jiang *et al.*, 2010; Bassil and Blumwald, 2014).

The ability to respond to high salinity via changes in the expression of the V-ATPase subunits encoding genes might be a prerequisite and a characteristic of salt stress tolerance in plants. It has been reported that the transcript levels of some subunits are up-regulated in response to high salinity (Narasimhan *et al.*, 1991; Kirsch *et al.*, 1996; Silva and Gerós, 2009). However, the expression of V-ATPase genes does not always involve a fixed stoichiometry of mRNAs for the different subunits (Silva and Gerós, 2009). Other factors may also account for the regulation of V-ATPase activity. For instance, the VHA-A subunit from barley (*Hordeum vulgare* L.) was shown to interact to 14-3-3 proteins, well known activators of PM ATPases, in a phosphorylation-dependent way. That interaction was suggested to activate V-ATPase activity (Klychnikov *et al.*, 2007).

### Plasma membrane and vacuolar H^+^-PPase

H^+^-pyrophosphatases (H^+^-PPase) are highly hydrophobic single subunit proteins that generate proton gradient across the vacuole, Golgi and plasma membrane using the energy of hydrolysis of pyrophosphate (PPi) molecules (Gaxiola *et al.*, 2007). Plants have two phylogenetically distinct types of H^+^-PPases: type I and type II. Type I H^+^-PPases depend on cytosolic K^+^ for their activity and are moderately sensitive to inhibition by Ca^2+^, and type II H^+^-PPases are K^+^ insensitive but extremely Ca^2+^ sensitive.

The Arabidopsis genome encodes two H^+^-PPases: a type I H^+^-PPase (AVP1) and a type II H^+^-PPase (AVP2) (Drozdowicz *et al.*, 2000). The rice genome also encodes two H^+^-PPases: OVP1 and OVP2 (Sakakibara *et al.*, 1996). However, more isoforms have been proposed (Choura and Rebai, 2005). Phylogenetic analysis of V-PPase sequences showed that rice H^+^-PPases are likely to be type I H^+^-PPases (Drozdowicz *et al.*, 2000). Type I H^+^-PPases are mainly suggested to acidify the vacuole (Gaxiola *et al.*, 2007). However these H^+^-pumps were also found in the plasma membrane (Ratajczak *et al.*, 1999; Alexandersson *et al.*, 2004). Arabidopsis type II H^+^-PPase, AVP2, has been shown to localize exclusively to Golgi apparatus (Mitsuda *et al.*, 2001).

The expression levels of the H^+^-PPases are strictly regulated at transcriptional level in response to various environmental conditions or developmental stages. It has been shown that the pollen-specific *cis*-acting region of the *AVP1* gene is involved in the regulation of the gene expression during pollen development. AtCAM15, AtCAMTA 1 (calmoduline-binding transcription factors) (Mitsuda *et al.*, 2003), AtVOZ1, and AtVOZ2 (*Arabidopsis thaliana* Vascular plant One Zinc finger protein) (Mitsuda *et al.*, 2004) were identified as binding to the *cis*-acting region of the *AVP1* gene (Silva and Gerós, 2009; Fuglsang *et al.*, 2010). Salt stress was reported to increase H^+^-PPase activity (Maeshima, 2000). However, a comprehensive mechanism of H^+^-PPase gene expression and post-translational regulation is still needed. It is likely that the protein C-terminus plays an essential role in supporting the physiological function of H^+^-PPase activity (Fuglsang *et al.*, 2010).

Given the importance of the pH homeostasis in the cytosol for cell metabolism, it is likely that the activity of all three H^+^-pumps (P-ATPase, V-ATPase and H^+^-PPase) is regulated by common regulatory mechanisms. 14-3-3 proteins, which are known to regulate many membrane localized proteins, particularly cell ion pumps (Bunney *et al.*, 2002), may be involved in such mechanisms.

## SOS1 and the plant response to salt stress

Comparisons of unidirectional Na^+^ fluxes and rates of net accumulation of Na^+^ in root indicate that 70-99% of the Na^+^ transported into the root is extruded back to the apoplast (Munns, 2005; Tester and Davenport, 2003). For rice, that value is indicated as 96% (Munns, 2005), meaning that over time Na^+^ will accumulate in roots and being transferred via the transpiration stream to the shoot, later accumulating there. Since it is important to maintain low cytoplasmatic Na^+^ concentrations for growth and survival under saline conditions, plants have developed a direct mechanism to extrude Na^+^ from cells across the plasma membrane to the soil or apopoplast. Small differences in Na^+^ exclusion capacity create major changes in Na^+^ net accumulation (Tester and Davenport, 2003; Munns, 2005; Brini and Khaled., 2012). However, the role of cellular Na^+^ efflux is not intuitive in multicellular plants, as Na^+^ transport out of one cell would negatively impact the surrounding neighbor cells. So, the role of Na^+^ efflux has to be considered in specific tissues and in the context of the whole plant (Zhu, 2003). Sodium efflux is catalyzed by the plasma membrane Na^+^/H^+^ antiporter encoded by *SOS1* (*Salt Overly Sensitive1* = *AtNHX7*) gene, identified in several plants including Arabidopsis (Wu *et al.*, 1996), rice (Martínez-Atienza *et al.*, 2007), wheat (Xu *et al.*, 2008), and tomato (Xu *et al.*, 2008). SOS1 uses the proton gradient established by P-ATPase and/or plasma membrane H^+^-PPase to exchange Na^+^ for H^+^ across the membrane (Shi and Zhu, 2002; Qiu *et al.*, 2004; Ji *et al.*, 2013). Activity of the Arabidopsis *SOS1* promoter is detected ubiquitously in virtually all tissues, but it appears to be more active in: (1) root epidermal cells (particularly at the root tip), suggesting that meristem requires special protection, since the root tip cells have very small vacuoles and thus are incapable of vacuolar Na^+^ compartmentalization, and (2) root parenchyma cells lining the vasculature (Shi and Zhu, 2002; Kronzucker and Britto, 2011). The *SOS1* gene expression pattern, together with the results of ion analysis in *sos1* mutant plants, suggest that SOS1 has several roles: (1) Na^+^ efflux from roots; (2) slowing down Na^+^ accumulation in the cytoplasm in order to gain time for Na^+^ storage in the vacuole; and (3) control of long-distance Na^+^ transport between roots and leaves by loading and unloading Na^+^ into and from the xylem (Zhu, 2003; Conde *et al.*, 2011). SOS1 may mediate active loading of Na^+^ to the xylem under mild salinity (25 mM NaCl). However, at high salinity (100 mM NaCl), expression of *SOS1* is induced and SOS1 may function in Na^+^ retrieval from the xylem (Shi *et al.*, 2002). Such a role for SOS1 in long-distance transport is important for the coordination between transpiration Na^+^ flow and Na^+^ vacuolar sequestration in leaves. However, a thermodynamic analysis by Munns and Tester (2008) indicated that the Na^+^ removal from the xylem is unlikely to be mediated by a Na^+^/H^+^ antiporter such as SOS1, because its operation “in reverse” under high Na^+^ conditions is thermodynamically unfavorable. Instead, class I HKTs have been shown to be involved in xylem unloading of Na^+^ (Ren *et al.*, 2005; James *et al.*, 2006; Davenport *et al.*, 2007). Thus, the role of SOS1 in long-distance Na^+^ transport remains unclear. Nevertheless, many reports suggest that SOS1 plays a critical role in Na^+^ exclusion, thus maintaining cellular ion homeostasis and allowing plants to survive and grow under salt stress conditions (Shi *et al.*, 2003; Cuin *et al.*, 2011) (Table 1).

**Table 1:** List of NHX antiporters including information about species, transport selectivity, tissue localization, sub-cellular localization and plant function for each NHX antiporter described in this review. No information available (N/A), Plasma membrane (PM), *trans*-Golgi network (TGN), and prevacuolar compartment (PVC).

Transporter	Species	Transport selectivity	Tissue localization	Subcellular localization	Function in planta	Refs.
AtNHX1	Arabidopsis	Na^+^/K^+^	Roots: Vascular tissues Shoots: Floral and vascular tissues, guard cells, trichome.	Tonoplast	K^+^ homeostasis and pH regulation	Rodríguez-Rosales *et al.*, 2009; Bassil *et al.*, 2011b; Yokoi *et al.*, 2002
AtNHX2	Arabidopsis	Na^+^/K^+^	Roots Shoots: High in guard cells	Tonoplast	K^+^ homeostasis and pH regulation	
AtNHX3	Arabidopsis	Na^+^/K^+^	Mainly in roots	Tonoplast	N/A	
AtNHX4	Arabidopsis	Na^+^/K^+^	Shoots: Mainly in mature pollen and seeds	Tonoplast	N/A	
AtNHX5	Arabidopsis	Na^+^/K^+^	Roots Shoots: High in guard cells	TGN, PVC	pH homeostasis in TGN, PVC	
AtNHX6	Arabidopsis	Na^+^/K^+^	Roots Shoots: High in guard cells	TGN, PVC	pH homeostasis in TGN, PVC	
AtNHX7/SOS1	Arabidopsis	Na^+^	Roots: Epidermal cells (particularly root tip), parenchyma cells lining the vasculature Shoots	PM	Na^+^ efflux	Martínez-Atienza *et al.*, 2007; Shi and Zhu, 2002; Kronzucker and Britto, 2011
AtNHX8	Arabidopsis	N/A	N/A	PM	N/A	N/A
OsNHX1	Rice	Na^+^/K^+^	Roots: Stela, emerging parts of lateral roots. Shoots: Basel part of seedling shoot, vascular bundle, flag leaf sheaths, panicles, guard cells, trichome.	Tonoplast	N/A	Fukada *et al.*, 2004; Fukada *et al.*, 2011; Bassil *et al.*, 2012
OsNHX2	Rice	Na^+^/K^+^	Shoots: Flag leaf sheaths, panicles.	Tonoplast	N/A	
OsNHX3	Rice	Na^+^/K^+^	Shoots: Flag leaf sheaths, panicles.	Tonoplast	N/A	
OsNHX4	Rice	N/A	N/A	Tonoplast	N/A	
OsNHX5	Rice	Na^+^/K^+^	Roots: Stela, emerging parts of lateral roots, root tip. Shoots: Basel part of seedling shoot, vascular bundle, flag leaf sheaths, panicles, pollen grain.	TGN, PVC	N/A	
OsNHX7/OsSOS1	Rice	Na^+^	Roots and shoots	PM	Na^+^ efflux	Martinéz-Atienza *et al.*, 2007

The transcript level of *SOS1* is upregulated by high salinity (Shi *et al.*, 2000). Analysis of the 2 Kb upstream of the *SOS1, CIPK24/SOS2* and *CBL4/SOS3* transcription initiation sites revealed that the promoter of these genes contains several binding elements for transcription activation of the bZIP, NAC, WRKY, and TCP classes (Ji *et al.*, 2013). However, transcription factors (TFs) mediating promoter activity of SOS genes have not yet been identified. Up-regulation of *SOS1* transcript levels under high salinity is suggested to be regulated at the post-transcriptional level, as *SOS1* promoter activity is not up-regulated by salt stress, but the *SOS1* gene expression driven by the constitutive Cauliflower mosaic virus 35S promoter is (Shi *et al.*, 2003). This may indicate that the *SOS1* transcript is unstable in the absence of salt stress and that the salt stress causes a post-transcriptional stabilization of the transcript (Shi *et al.*, 2003). More recently, it was suggested that the Na^+^ stress induced *SOS1* mRNA stability is mediated by ROS (Chung *et al.*, 2008). In addition, regulation of *SOS1* transcript levels by high salinity is partly under the control of SOS2 and SOS3 (Shi *et al.*, 2000). CIPK24/SOS2 is a protein kinase and CBL4/SOS3 is a calcium sensor that, together with SOS1, are the three key components comprising the Salt Overlay Sensitive (SOS) signaling pathway identified in Arabidopsis (Wu *et al.*, 1996) and rice (Martínez-Atienza *et al.*, 2007). At the cellular level, the SOS signaling pathway has been proposed to mediate cellular signaling under salt stress to maintain the ion homeostasis (Ji *et al.*, 2013).

Activation of the Na^+^/H^+^ antiport activity of SOS1 by salt stress is controlled by SOS3 and SOS2 (Zhu, 2003; Ji *et al.*, 2013). In response to an external stimulus, such as high Na^+^ concentration, transient increases in cytoplasmatic Ca^2+^ occur and that is decoded by the calcineurin B and neuronal Ca^2+^ sensor-like protein SOS3. Activation of SOS3 requires N-myristoylation and Ca^2+^ bound on EF-hand Ca^2+^ binding sites. Activated SOS3 physically interacts with the auto-inhibitory domain of SOS2, a member of the SnRK (sucrose non-fermenting-related serine/threonine kinase) family, which activates the kinase and facilitates the localization of the SOS2-SOS3 complex to the plasma membrane. The SOS2-SOS3 complex associates with the Na^+^/H^+^ antiporter SOS1, phosphorylating its C-terminal auto-inhibitory domain, which becomes activated and thus pumps Na^+^ out of the cell (Pardo, 2010; Brini and Khaled, 2012; Hasegawa, 2013; Ji *et al.*, 2013) (Figure 2).

The SOS pathway is not limited to the three main proteins, as it interacts with other stress related proteins. A SOS3 homolog SOS3-LIKE Calcium Binding Protein8 (SCABP8/CBL10) interacts with SOS2 to form an alternative protein kinase complex that regulates SOS1 activity in the plasma membrane in response to salt stress, mainly in shoots, while SOS3 functions primarily in the root (Quan *et al.*, 2007) (Figure 2). SOS2 phosphorylates CBL10 in a Ca^2+^ independent manner upon salt stress, and this phosphorylation stabilizes the SOS2-CBL10 complex association with the plasma membrane and increases SOS1 antiporter activity (Kim *et al.*, 2007; Quan *et al.*, 2007; Hasegawa, 2013). Abscisic acid insensitive 2 (ABI2) interacts with SOS2 to prevent the SOS3 binding to SOS2 and kinase activation. Such ABI2-SOS2 interaction may represent an integrating node between salt stress and ABA signaling (Ohta *et al.*, 2003; Hasegawa, 2013).

The SOS pathway may also regulate the Na^+^ vacuolar compartmentalization. Interaction of SOS2-CBL10 may result in localization of the kinase complex at the vacuolar membrane where it is possibly involved in the regulation of Na^+^/H^+^ exchange at the tonoplast, presumably by regulation of NHX antiporter(s) activity (Qiu *et al.*, 2004; Kim *et al.*, 2007). However, no NHX antiporter has already been shown to be directly regulated by SOS2 and/or by the SOS2-complex. In addition, SOS2 has been suggested to regulate the V-ATPase activity. SOS2 was found to interact with the B1 and B2 subunits of the V-ATPase in the absence of CBL proteins, and tonoplast vesicles from the Arabidopsis *sos2-2* mutant showed reduced ATPase and H^+^-translocation activities (Batelli *et al.*, 2007).

Potassium homeostasis has also been shown to be modulated by the SOS signaling pathway. The protein CBL10 has been indicated to directly interact with AKT1 channel and negatively regulate its activity in roots (Ren *et al.*, 2013). It is well known that plant salt stress tolerance is closely related to maintenance of high K^+^/Na^+^ cytosolic ratio under stress (Tester and Davenport, 2003). The possibility that CBL10 functions as an interconnecting regulator of SOS1 and AKT1 may indicate that CBL10 plays a crucial role in ion homeostasis (K^+^/Na^+^) under salt stress by regulating both K^+^ and Na^+^ uptake/exclusion (Ren *et al.*, 2013).

## HKTs and the plant responses to salt stress

Another important determinant of salt stress tolerance in plants is the activity of the HKT (high affinity potassium transporter) proteins (Munns and Tester, 2008; Roy *et al.*, 2014). The HKT family is quite diverse, and this diversity reflects their large amplitude of functions (Munns and Tester, 2008; Almeida *et al.*, 2013; Roy *et al.*, 2014). The HKT family is divided in two distinct classes according to their transport characteristics. The main distinguishing feature is the amino acid sequence that constitutes the first pore domain (PD) (Platten *et al.*, 2006). Members of class I transporters (HKT1) have a serine (S), forming an S-G-G-G motif, where most of the members of class II (HKT2) have a G in the position occupied by the S in class I transporters, forming a G-G-G-G motif (Maser *et al.*, 2002). The presence of either S or G at this position is critical for the cation specificity of transporter. The presence of an S (HKT1) is characterized by a preference for Na^+^ conductance over other cations, whereas the presence of a G (HKT2) is characterized by transport of Na^+^ and/or K^+^ depending on the external concentrations of these two ions (Platten *et al.*, 2006; Kronzucker and Britto, 2011). However, there are notable exceptions, in particular HKT2;1 from cereals, in which the G has reverted to S (Kronzucker and Britto, 2011), but it has been clearly shown to be involved in mediating Na^+^ and K^+^ entry into roots (Munns and Tester, 2008; Kronzucker and Britto, 2011). The main role of HKT1 is believed to be Na^+^ retrieval from the transpiration stream avoiding the over accumulation of Na^+^ in the photosynthetic tissues.

### HKT1 family

The best characterized member of HKTs class I is AtHKT1;1 from Arabidopsis. Disruption of *AtHKT1;1*, the only member of HKT family in Arabidopsis, caused a higher accumulation of Na^+^ in the shoots but reduced concentration in roots, with little effect on the net Na^+^ uptake (Rus *et al.*, 2004; Pardo, 2010; Kronzucker and Britto, 2011) *AtHKT1;1* is preferentially expressed in the plasma membrane of xylem parenchyma cells and phloem cells of both roots and leaves, where it is suggested to regulate the Na^+^ distribution between roots and shoots (Sunarpi *et al.*, 2005; Moller *et al.*, 2009; Pardo, 2010; Kronzucker and Britto, 2011) (Figure 1 and Table 2). Two complementary functions for AtHKT1;1 have been proposed. In the phloem recirculation model, AtHKT1;1 loads Na^+^ into shoot phloem cells to be transferred to roots via the downward stream, preventing Na^+^ overaccumulation in the shoot. However, the overall Na^+^ retranslocation potential via phloem should not exceed 10% of the total Na^+^ loaded in the shoot xylem transpiration stream (Berthomieu *et al.*, 2003). Another function of AtHKT1;1 is to unload Na^+^ from the xylem transpiration stream, thereby restricting the amount of Na^+^ reaching the photosynthetic tissues and supporting salt stress tolerance.

**Table 2:** List of HKT transporters including information about class, species, transport selectivity, tissue localization, subcellular localization and plant function for each HKT transporter described in this review. No information available (N/A), Plasma membrane (PM).

Transporter	Species	Transport selectivity	Tissue localization	Subcellular localization	Function in planta	Refs.
**Class I**						
AtHTK1;1	Arabidopsis	Na^+^	Roots: Xylem parenchyma, phloem Shoots: Phloem	PM	Unload Na^+^ from the xylem transpiration stream Load of Na^+^ into shoot pholem	Berthomieu *et al.*, 2003; Møller *et al.*, 2009; Sunarpi *et al.*, 2005
OsHTK1;1	Rice	Na^+^	Roots: Similar as OsHKT2;1 Leaves: Bulliform cells and vascular tissues	PM	N/A	Jabnoune *et al.*, 2009
OsHTK1;2	Rice	N/A	N/A	PM	N/A	Wu *et al.*, 2009
OsHTK1;3	Rice	Na^+^	Roots: Cortex and vascular tissues in the stele. Leaves: Bulliform cells and vascular tissues, mesophyll cells.	PM	N/A	Jabnoune *et al.*, 2009
OsHTK1;4	Rice	N/A	Leaves sheaths	PM	Control of sheath to blade transfer of Na^+^	Cotsaftis *et al.*, 2012
OsHTK1;5	Rice	Na^+^	Roots and Shoots: Xylem parenchyma	PM	Unload Na^+^ from the xylem transpiration stream	Ren *et al.*, 2005
TaHKT1;4	Wheat	Na^+^	Roots and Leaves	PM	Unload Na^+^ from the xylem transpiration stream	Huang *et al.*, 2006
TaHKT1;5	Wheat	Na^+^	Roots	PM	Unload Na^+^ from the xylem transpiration stream	Byrt *et al.*, 2007;Munns *et al.*, 2012
**Class II**						
OsHKT2;1	Rice	Na^+^/K^+^	Roots: Epidermis, exodermis, cortex differentiated into aerenchyma, stele (mainly pholem). Leaves: Bulliform cells, xylem, phloem, mesophyll cells.	PM	Nutritional Na^+^ uptake from the external medium.	Jabnoune *et al.*, 2009; Horie *et al.*, 2007
OsHKT2;2	Rice	Na^+^/K^+^	Roots	PM	Na^+^ and K^+^ uptake under K^+^ starvation conditions.	Yao *et al.*, 2010; Oomen *et al.*, 2012
TaHKT2;1	Wheat	Na^+^/K^+^	Roots: Cortical and stele Leaves: Vasculature tissue of mesophyll.	PM	Na^+^ uptake from external medium.	Schachtman and Schroeder, 1994
HvHKT2;1	Barley	Na^+^/K^+^	Roots: Cortex. Leaves: Blade and sheath.	PM	K^+^ uptake at very low K^+^ concentrations (possible) and uptake of Na^+^ in the roots (possible).	Mian et a., 2011; Haro *et al.*, 2005

Analysis of several QTLs of salt tolerance in rice (Ren *et al.*, 2005; Kronzucker and Britto, 2011) and wheat (James *et al.*, 2006; Byrt *et al.*, 2007; Kronzucker and Britto, 2011) has provided further evidence for the importance of *HKT* class 1 genes in controlling Na^+^ accumulation in leaves upon salt stress. In rice, QTL analyses showed that higher shoot K^+^ content of the salt-tolerance *indica* genotype, Nona Bokra, cosegregated with an allelic variant of *SKC1* (Shoot K^+^ Content 1) with higher activity as compared to that of the salt-sensitive *japonica* genotype, Koshihikari (Ren *et al.*, 2005). SCK1, now referred to as *OsHKT1;5* (*OsHKT8*) is a plasma membrane, K^+^ independent, and Na^+^ selective transporter that is preferentially expressed in the parenchyma cells surrounding xylem vessels (Ren *et al.*, 2005; Pardo, 2010; Almeida *et al.*, 2013) (Table 2). The Nona Bokra *OsHKT1;5* has four amino acids different from the Koshihikari protein, and this difference has been associated with greater Na^+^ transporter activity and increased ability for maintenance of K^+^/Na^+^ homeostasis under salt stress (Ren *et al.*, 2005). Rice contains four more HKT1 members in the genome, *OsHKT1;1, OsHKT1;2, OsHKT1;3, OsHKT1;4* (Ren *et al.*, 2005; Huang *et al.*, 2006; Wu *et al.*, 2009; Cotsaftis *et al.*, 2012; Almeida *et al.*, 2013) (See Table 2 for further information). *OsHKT1;4* gene expression is up-regulated in the leaf sheaths under salt stress (Cotsaftis *et al.*, 2012) and encodes three different splicing forms, identified in both rice genotypes Pokkali (salt-tolerant) and Nipponbare (salt-susceptible). All *OsHKT1;4* splicing forms are translated into protein, nevertheless only the longer splicing form seems to be translated into a functional protein (Cotsaftis *et al.*, 2012). Interestingly, Pokkali is able to maintain a much higher ratio of functional *OsHKT1;4* transcripts in younger leaf sheaths as compared to Nipponbare. In addition, transcript levels of the functional transcripts were inversely correlated with the individual leaf blade Na^+^ concentration in both genotypes (Cotsaftis *et al.*, 2012). At this point it seems that the longer *OsHKT1;4* splicing form is the key transporter controlling the sheath-to-blade transfer of Na^+^ in rice shoots (Cotsaftis *et al.*, 2012).

In wheat, QTL analyses using durum wheat (*Triticum turgidum* L. subsp. *durum*) breeding Line 149 led to the identification of two loci, *Nax1*, and *Nax2*, which decreased Na^+^ accumulation in the leaf blade (James *et al.*, 2006; Byrt *et al.*, 2007; Kronzucker and Britto, 2011). In addition, bread wheat (*Triticum aestivum*), which is an allohexaploid (2n = 6s = 42, genome AABBDD), was found to be more salt tolerant than the allotetraploid pasta wheat (AABB genomes). It was shown that the D genome carries a locus (*Kna1*) responsible for maintenance of high K^+^/Na^+^ ratio during salt stress justifying the salt tolerance of bread wheat (Dubcovsky *et al.*, 1996; Byrt *et al.*, 2007; Kronzucker and Britto, 2011). The process controlled by the *Nax2* and *Kna1* loci reduces net root xylem loading of Na^+^, while the *Nax1* locus reduces Na^+^ accumulation in the leaf blade by restricting Na^+^ loading into root xylem and partitioning Na^+^ into the leaf sheath (James *et al.*, 2006; Hasegawa, 2013). Using high-resolution mapping, *Nax1* and *Nax2* were identified as members of the *HKT1;4* gene family and *Kna1* as member of the *HKT1;5* gene family (Table 2). Because both *Nax* genes are originated from a wheat relative, *Triticum monococcum*, that was crossed with a durum wheat, they were named *TmHKT1;4-A2* and *TmHKT1;5-A*, respectively. The *Nax2* region of the breeding Line 149 was found to correspond to the *Kna1* region of the bread wheat and *Kna1* was named *TaHKT1;5-D* (Almeida *et al.*, 2013). *Nax1* and *Nax2* genes do not exist in modern bread or durum wheat genotypes, and introgression of *Nax1* or *Nax2* into bread wheat led to reduced leaf blade Na^+^ accumulation and increased leaf blade Na^+^ exclusion relative to the parent respectively. The combination of *Nax1* and *Nax2* further decreased Na^+^ accumulation in the leaf blade (James *et al.*, 2011), showing that these genes clearly have similar functions as *AtHKT1;1* in Arabidopsis and *OsHKT1;5* and *OsHKT1;4* in rice (Ren *et al.*, 2005; James *et al.*, 2006; Almeida *et al.*, 2013) (Table 2). Moreover, field trials with durum wheat, carrying the *Nax2* gene, growing under high saline soils showed a 25% increase in grain yield and reduced Na^+^ accumulation in flag leaf as compared to a near isogenic line without the *Nax2* locus (Munns *et al.*, 2012). Altogether, these results indicate that *HKT1* mediated Na^+^ exclusion from shoot is an effective mechanism for enhancing salt stress tolerance in crop plants.

Concerning, *HKT1* transcriptional regulation, some transcriptional regulatory elements have been identified in the *AtHKT1* promoter. The tandem repeat regions (R1 and R2) found in the distal *AtHKT1* promoter region located about 3.9 kb upstream of the translational start codon were responsible for expression of *AtHKT1* in roots (Rus *et al.*, 2004; Baek *et al.*, 2011). The repeat sequence R2 which is closest to ATG acts as an enhancer element of *AtHKT1* expression. Its inactivation caused reduced *AtHKT1* expression in root and higher Na^+^ accumulation in shoot (Rus *et al.*, 2004; Baek *et al.*, 2011). The *AtHKT1* promoter contains a highly methylated GC region (250 bp) at 2.6 kb upstream of the translational start codon. Interestingly, methylation in the leaf is higher than in roots, which suggests that higher methylation in this promoter region is required to maintain *AtHKT1* expression at low levels and perhaps in a correct pattern of expression in the different tissues (Baek *et al.*, 2011). Furthermore, this region contains a putative small RNA target site, which was suggested to be involved in methylation guided by small RNAs (Baek *et al.*, 2011).

### HKT2 family

HKT class 2 proteins are generally found in monocotyledonous species, and no HKT class 2 homologs have been identified in dicotyledonous species (Platten *et al.*, 2006; Adams and Shin, 2014). Four HKT class 2 members have been characterized in detail: OsHKT2;1 and OsHKT2;2 in rice, TaHKT2;1 in wheat, and HvHKT2,1 in barley (*Hordeum vulgare* L.) (Table 2). These transporters have common properties thought to be shared by all HKT2 class 2 members, such as a role in Na^+^ uptake from external medium under K^+^ limiting conditions (Almeida *et al.*, 2013).

The two characterized rice members of this HKT family, OsHKT2;1 and OsHKT2;2, have been reported to mediate Na^+^ uptake from soil under K^+^ limiting conditions (Table 2). *OsHKT2;1* gene expression is induced by K^+^ deficiency (Horie *et al.*, 2001; Yao *et al.*, 2010). OsHKT2;1 is an atypical HKT class 2 member, which has an S residue in the first PD and mediates high-affinity Na^+^ uptake. However, OsHKT2;1 can also mediate K^+^ transport depending on the external concentration of both K^+^ and Na^+^ (Jabnoune *et al.*, 2009; Yao *et al.*, 2010; Almeida *et al.*, 2013). OsHKT2;1 is known to be highly involved in “nutritional” absorption of Na^+^ and its relevance in Na^+^ uptake during salt stress may be limited since it has a micromolar affinity for Na^+^ and its activity is rapidly downregulated at high Na^+^ concentration. Interestingly, RNA levels of at least three other *OsHKTs* genes have been shown to be inhibited by an external Na^+^ concentration as low as 30 mM (Horie *et al.*, 2001). On the other hand, OsHKT2;2 has only been found in the salt-tolerant Nona Bokra and Pokkali genotypes, being absent in the rice salt-sensitive Nipponbare genotype, which suggests that the presence of OsHKT2;2 is an evolutionary advantage for salt-tolerant genotypes (Horie *et al.*, 2001; Almeida *et al.*, 2013). OsHKT2;2 is expressed in roots among other tissues and transporting both K^+^ and Na^+^, but under salinity only Na^+^ is transported (Kader *et al.*, 2006, Oomen *et al.*, 2012) (Table 2). Other OsHKT class 2 members have also been identified (OsHKT2;2/1, OsHKT2;3, OsHKT2;4), however these will be not described. For further information, see Almeida *et al.* (2013) and Kronzucker and Britto (2011).

In wheat, TaHKT2;1 seems to have a function in root Na^+^ influx similar to rice OsHKT2;1 (Horie *et al.*, 2009). *TaHKT2;1* is expressed in the root cortex and is induced by K^+^ deficiency (Schachtman and Schroeder, 1994). *In planta*, TaHKT2;1 has been suggested to have a role in Na^+^ transport with a possible role in root Na^+^ uptake, though TaHKT2;1 was also reported to transport K^+^ (Almeida *et al.*, 2013) (Table 2).

In barley, a relative salt-tolerant species, *HvHKT2;1,* is preferentially expressed in root cortex and to a much lower level in leaf blade and sheaths, and it is induced by K^+^ deficiency in roots and shoots and by high Na^+^ concentration in shoots. *HvHKT2;1* mediates both K^+^ and Na^+^ transport (Haro *et al.*, 2005; Mian *et al.*, 2011; Almeida *et al.*, 2013) (Table 2). Transgenic barley lines over-expressing *HvHKT2;1* result in higher Na^+^ concentration in xylem, enhanced translocation of Na^+^ to shoots and Na^+^ accumulation in the leaves higher than in the non-transformed plants, supporting the hypothesis that this transporter is able to mediate root Na^+^ uptake (Mian *et al.*, 2011). Moreover, transgenic plants showed a significant increase in shoot K^+^ content in plants growing in limiting K^+^ conditions, suggesting that *HvHKT2;1* may also play a role in K^+^ absorption or re-absorption at very low K^+^ concentrations (Mian *et al.*, 2011).

## NHX and the plant response to salt stress

At the cellular level, high amounts of Na^+^ can be tolerated by intracellular partitioning so that the concentration in the cytoplasm is kept as low as 10-30 mM (Munns and Tester, 2008). This strategy can be used by plants for the alleviation of excessive cytosolic Na^+^ by sequestrating Na^+^ into the vacuole, which typically makes up to 80-90% of the cell volume. Other organelles, such as endosomal compartments, plastids and mitochondria, may also accumulate Na^+^ and thus contribute to the overall subcellular Na^+^ sequestration (Zhu, 2003). The vacuolar sequestration of Na^+^ that occurs in all tissues is not only important for Na^+^ detoxification in the cytosol, but it is also a critical mechanism of osmotic adjustment to maintain water uptake from saline solutions (Zhu, 2003; Munns and Tester, 2008; Bassil *et al.*, 2012).

An increased vacuolar Na^+^ concentration requires a coordinated increase in the osmotic pressure of the other subcellular components, including the cytosol, to maintain the osmotic pressure and thereby the volume. This can be achieved by an increase in the K^+^ concentration to a sub-toxic level, as well as by the synthesis and accumulation of compatible solutes (*e.g.*, proline, sucrose, glycine betaine, etc.). Nevertheless, the latter represents a major drawback due to the high energetic cost associated with solute synthesis (Munns and Tester, 2008; Maathuis *et al.*, 2014).

The tonoplast controls the movement of inorganic and organic solutes to and from the cytoplasm through a wide range of pumps, carriers and ion channels (Conde *et al.*, 2011). Cation/H^+^ antiporters mediate the transport of Na^+^ into the vacuole, driven by the electrochemical gradient of protons generated by the V-ATPase and V-PPase enzymes (Jiang *et al.*, 2010; Bassil and Blumwald, 2014). This Na^+^/H^+^ exchange is mediated by members of a family of transporters referred to as Na^+^/H^+^ antiporters (NHXs) in plants or Na^+^/H^+^ exchange (NHEs) in animals (Jiang *et al.*, 2010; Bassil and Blumwald, 2014). In addition, plant NHX antiporters mediate both Na^+^/H^+^ and K^+^/H^+^ exchange, therefore affecting both salinity tolerance and K^+^ nutrition (Venema *et al.*, 2002; Leidi *et al.*, 2010).

## Diversity of plant NHX antiporters

Plant NHX proteins belong to a large superfamily of monovalent cation/proton antiporters (CPAs) made up of two subgroups, CPA1 and CPA2. The CPA2 family includes members of the less known Cation/H^+^ Exchangers (CHXs) and K^+^ efflux antiporters (KEA). The CPA1 family includes members of the NHX-type, which are ubiquitous in all eukaryotic organisms (Rodríguez-Rosales *et al.*, 2009; Bassil *et al.*, 2012). In Arabidopsis, NHX-type antiporter family members comprise eight members that are divided into two distinct classes; two divergent members located at the plasma membrane (SOS1/AtNHX7 and AtNHX8), and six intracellular members located either at the tonoplast (AtNHX1-AtNHX4) or the endosomal membrane (Golgi, *trans*-Golgi network and prevacuolar compartments) (Rodríguez-Rosales *et al.*, 2009; Bassil *et al.*, 2012; Reguera *et al.*, 2015). In rice, six NHX-type antiporter family members were identified as belonging to two distinct classes with different cellular localizations: one in the plasma membrane (SOS1) (Martínez-Atienza *et al.*, 2007), and five intracellular members that are either in the tonoplast, OsNHX1 to OsNHX4, or in the prevacuolar compartment OsNHX5 (Fukuda *et al.*, 2011) (Figures 1 and 2, Table 1). In Arabidopsis, the most abundant members of NHX-types are AtNHX1 and AtNHX2, accounting for a significant amount of the K^+^-Na^+^/H^+^ antiport activity in tonoplast vesicles (Barragan *et al.*, 2012). Detailed information regarding AtNHXs and OsNHXs tissue localization is described in Table 1.

## *NHX* gene expression under stress conditions

In Arabidopsis seedlings, *AtNHX1* and *2* were shown to be induced by salt stress (NaCl), hyperosmotic stress (mannitol) and ABA treatment, whilst *AtNHX5* was only induced by salt stress (NaCl) (Yokoi *et al.*, 2002). In rice seedlings, salt stress (NaCl), hyperosmotic stress (mannitol) and ABA treatment increased the transcript levels of *OsNHX1, 2, 3* and *5* (Fukuda *et al.*, 2011). These reports show that *NHX* genes are components of the plant salt stress response. Interestingly, treatment with a high KCl concentration induced the expression of *OsNHX1* and *2* (Fukuda *et al.*, 2011), and *AtNHX1* (Yokoi *et al.*, 2002). *AtNHX1* and *2* were induced by ABA but not by NaCl in the ABA-deficient *aba2-1* mutant, showing that NaCl induction of these members depends on ABA signaling (Yokoi *et al.*, 2002). *AtNHX1* and *2* promoter sequences do not have ABA-responsive elements (ABRE). Nevertheless, the promoter of each gene contains MYC/MYB *cis*-regulatory elements, suggesting that *AtNHX1* and *2* are outputs of the ABA-dependent pathway regulated by these transcription factors (Yokoi *et al.*, 2002). On the other hand, the *OsNHX1* promoter (up to 1.8 kb upstream of the translational start codon) shows several ABA-responsive elements (ABRE), as well as drought responsive elements MYC/MYB *cis*-regulatory elements (Almeida *et al.*, unpublished results), indicating that *OsNHX1*, similar to *AtNHX1*, is also transcriptionally regulated by an ABA-dependent pathway. Interestingly, the SOS pathway also appears to regulate the activity of vacuolar Na^+^/H^+^ antiporters (Yokoi *et al.*, 2002; Qiu *et al.*, 2004). The activity of AtNHX1 is possibly regulated through interaction with the protein kinase *SOS2* (Qiu *et al.*, 2004) (Figure 2). It was reported that the vacuolar Na^+^/H^+^ antiporter activity of Arabidopsis membrane vesicles was significantly reduced in vesicles obtained from *sos2* null mutants, as compared to wild type controls, and could be stimulated *in vitro* by the addition of activated SOS2 protein (Qiu *et al.*, 2004). The activity was further inhibited by AtNHX1 antibodies. However, phosphorylation of AtNHX1 by SOS2 was not shown. It was further shown that SOS2 also interacts with several V-ATPase subunits (Figure 2), and that vesicles isolated from *sos2* null mutants show a considerable lower V-ATPase acidification (Batelli *et al.*, 2007). Thus, comparisons of antiporter activity in vesicles from *sos2* mutant and wild type is complex, as the proton force motive that drives ion transport is not similar in both cases (Batelli *et al.*, 2007).

## NHX regulation and structural organization

### Transcriptional regulation

Although multiple functional studies on NHX-type proteins, especially NHX1, have been carried out, the details of how *NHX1* is transcriptionally regulated remain poorly explored. Adler *et al.* (2010) reported that NHX1 from the relatively salt-tolerant crop, sugar beet (*Beta vulgaris* L.) is regulated under salt stress by one or more MYB transcription factors, which could not be identified yet. Despite the importance of rice, only one study reported the identification of a TF interacting with the *OsNHX1* promoter. Using a chromatin immunoprecipitation assay, an *OsbZIP71* TF was identified by Liu *et al.* (2014) as directly binding to the *OsNHX1* promoter. It was shown that *OsbZIP71* gene expression was strongly induced by drought, polyethylene glycol (PEG), and ABA treatments, but repressed by salt treatment. Transgenic rice lines overexpressing *OsbZIP71* (p35S::*OsbZIP71*) showed improved tolerance to drought, salt and PEG-induced drought stresses, suggesting that *OsbZIP71* plays an important role in ABA-mediated drought and salt tolerance in rice (Liu *et al.*, 2014). However, the authors did not show whether the identified TF is relevant for *OsNHX1* activation under stress.

### Post-translational modifications

The cation selectivity of Arabidopsis NHX1 appears to be regulated by its C-terminal tail through the interaction with the vacuolar lumen-localized calmodulin-like protein 15 (AtCaM15), in a Ca^2+^ and pH-dependent manner (Yamaguchi *et al.*, 2003). Under control physiological conditions, when the vacuole pH is acidic (pH 5.5) and the Ca^2+^ concentration is high, AtCaM15 is bound to the AtNHX1 C-terminal tail, resulting in a higher K^+^/H^+^ exchange activity over Na^+^/H^+^ activity (Yamaguchi *et al.*, 2003). On the other hand, salt stress often causes alkalization of the vacuole, which reduces AtCaM15 binding to AtNHX1. This leads to an increased Na^+^/H^+^ exchange activity over K^+^/H^+^ activity and subsequent enhanced vacuolar Na^+^ sequestration (Yamaguchi *et al.*, 2003; Rodríguez-Rosales *et al.*, 2009). In addition, phosphoproteomic studies in Arabidopsis and rice suggested that NHX antiporters are regulated by phosphorylation (Whiteman *et al.*, 2008a,b). In rice, the vacuolar OsNHX3 was reported to be phosphorylated at residue S471 located in the C-terminus. The same residue is conserved among the three other rice vacuolar NHXs members (OsNHX1, 2 and 4) (Bassil *et al.*, 2012). Sequence comparison analyses between rice NHXs and Arabidopsis NHX3, 5 and 6 revealed that the Arabidopsis NHXs members contained the same S residue at a similar position (Bassil *et al.*, 2012). So far, the biological role of such post-translational modifications has not yet been functionally characterized. Negrão *et al.* (2013) identified a significant nonsynonymous mutation at OsNHX1, serine 477 to asparagine (S477N), present in the rice salt-susceptible genotypes IR 29 and IR 64, but also in the salt-tolerant genotype FL 478 (a recombinant inbred line derived from an IR 29 x Pokkali cross). The loss of an S residue can imply the loss of a putative phosphorylation site, and S477 sits in a cluster of S residues with high phosphorylation probability and is itself a potential phosphorylation target (Negrão *et al.*, 2013). This residue is located in the C-terminus of the OsNHX1 protein, and it was suggested by the authors that the nonsynonymous mutation may affect the phosphorylation of the OsNHX1 C-terminal possibly by SOS2, which in turn results in lower activation of OsNHX1 exchanger activity. However, further studies are needed to test this hypothesis.

### Topology

The crystallographic structure of NHXs antiporters is not yet available. Epitope tagging and protease protection assays applied to full length expressed AtNHX1 in a yeast heterologous system unveiled that AtNHX1 has nine transmembrane domains, with an additional three “buried” domains that do not entirely span the membrane (Yamaguchi *et al.*, 2003). This study also showed that the hydrophilic C-terminus is oriented to the vacuolar lumen, a feature that is strikingly different from the proposed cytosolic C-terminus orientation of animal NHEs (Yamaguchi *et al.*, 2003; Bassil *et al.*, 2012). However, another study performed by Sato and Sakaguchi (2005), using only protein fragments, showed that AtNHX1 contains eleven transmembrane domains and a cytosolic C-terminus, resembling an overall membrane topology of the human NHE. The C-terminus of NHX members is highly divergent, even among closely related members within the same species. Because the C-terminus regulates the antiporter activity, it has been suggested that divergent C-terminal sequences may constitute a novel way to differentially regulate individual members (Bassil *et al.*, 2012).

## Function of the NHX antiporters

### Salt tolerance

Na^+^/H^+^ exchange at the tonoplast is generally accepted to play a major role in plant salt stress tolerance. Several reports indicate that NHX overexpression (in homologous or heterologous systems) confer salt stress tolerance in a wide range of plant species. Constitutive overexpression of *AtNHX1* appears to increase salt stress tolerance significantly in yeast (Aharon *et al.*, 2003), Arabidopsis (Apse *et al.*, 1999), tomato (Zhang and Blumwald, 2001) and cotton (He *et al.*, 2005). Constitutive overexpression of various cereal NHX homologs has been also reported to improve the salt stress tolerance of Arabidopsis (Brini *et al.*, 2007), rice (Fukuda *et al.*, 2004; Zhao *et al.*, 2006) and wheat (Xue *et al.*, 2004). These results show the fundamental role of these proteins in plant salt stress tolerance and explain why they have been a major focus for genetic engineering (Jiang *et al.*, 2010; Bassil *et al.*, 2012). However, increased salt stress tolerance was not always associated with an increased vacuolar Na^+^ accumulation (Fukuda *et al.*, 2004; Rodríguez-Rosales *et al.*, 2008, 2009; Jiang *et al.*, 2010). Most of the characterized NHX members can transport both K^+^ and Na^+^, and may have similar Km for these substrates (Jiang *et al.*, 2010). This means that, unless the cytoplasmatic Na^+^ concentration is significantly higher than that of K^+^ (difficult to occur even under salt stress conditions), NHX exchangers mainly mediate K^+^/H^+^ exchange rather than Na^+^/H^+^ exchange (Jiang *et al.*, 2010; Maathuis *et al.*, 2014). Indeed, *nhx1*/*nhx2* double null mutants in Arabidopsis resulted in impaired vacuolar K^+^ accumulation, enhanced vacuolar Na^+^ uptake, and a salt (NaCl) insensitive phenotype, compared to wild-type (Barragan *et al.*, 2012). Thus, it is likely that the contribution of NHX-like protein to plant salt stress tolerance is the maintenance of K^+^ homeostasis rather than sequestration of Na^+^ into the vacuole (Maathuis *et al.*, 2014). Nevertheless, it is still not clear what are the primary ions being transported by NHX-like protein *in planta*. Given that NHX1 cation selectivity is regulated by interacting partners (AtNHX1 is regulated by CaM15), it is difficult to interpret the ion content results from plants overexpressing *AtNHX1*, and possibly other NHXs members, due to a possible shortage of interacting partners (Rodríguez-Rosales *et al.*, 2009).

### K^+^ homeostasis

Besides their key role in salt stress tolerance, at control growth conditions vacuolar NHX proteins have a key role in mediating K^+^/H^+^ exchange for turgor regulation and pH control. Potassium is an essential plant nutrient and the most abundant cation in plants, comprising up to 10% of plant dry matter. K^+^ is an important cofactor in many biosynthetic processes, and in the vacuole it plays key roles in cell volume regulation (Barragan *et al.*, 2012; Andrés *et al.*, 2014).

During grape berry (*Vitis vinifera* L.) development, high *VvNHX1* transcript levels during the véraison and post-véraison stages would indicate that the increase in vacuolar K^+^ accumulation, mediated by *VvNHX1* is needed for vacuolar expansion. This process is coupled with a rapid accumulation of sugars that drives water uptake to the berry and the concomitant berry size increase, typical of the post-véraison growth stage (Hanana *et al.*, 2007).

Genetic studies in Arabidopsis firmly demonstrate the importance of NHXs in the regulation of K^+^ and pH homeostasis (Rodríguez-Rosales *et al.*, 2008; Leidi *et al.*, 2010; Bassil *et al.*, 2011a; Barragan *et al.*, 2012). The Arabidopsis *AtNHX1* single knockout mutant displayed an altered phenotype under control growth conditions, including smaller cells, smaller leaves, and other developmental irregularities associated with altered K^+^ homeostasis, which was correlated with lower K^+^/H^+^ and Na^+^/H^+^ antiport activity (Apse *et al.*, 2003; Sottosanto *et al.*, 2004). *AtNHX2* knockout did not display any obvious growth phenotype, but mutants lacking both *AtNHX1* and *2* displayed a significant reduction in cell expansion in all tissues, especially in rapidly elongating organs such as flowers filaments and hypocotyls of etiolated seedlings, as compared to *Atnhx1* mutant or wild-type plants (Bassil *et al.*, 2011a). These plants displayed poor seed set because their filaments did not elongate enough to position the anther close to the stigma. Though these plants had non-dehiscent anthers, flowers could be artificially pollinated (Bassil *et al.*, 2011a). In root and leaf cells of the double mutant, the vacuolar K^+^ content was about one-third of that from wild-type cells. The double mutant was also highly sensitive to the addition of external K^+^ (*nhx1nhx2* mutant has higher K^+^ cytosolic content), which may indicate that these vacuolar NHX antiporters are the main mediators of cytosolic K^+^ uptake into the vacuole; it also suggests that variations of K^+^ supply, which would otherwise result in a fluctuation of cytosolic K^+^ content, is essentially buffered by vacuolar K^+^/H^+^ exchange, likely promoted by the activity of vacuolar NHX proteins (Bassil *et al.*, 2011a; Bassil and Blumwald, 2014). Impaired osmoregulation in the *nhx1nhx2* mutant leads to lower leaf water content, lower cell turgor and consequent defective stomatal movement. Altogether, it results in a poor plant water status maintenance (Barragan *et al.*, 2012). Stomatal movements rely on guard cell turgor and require massive bidirectional K^+^ fluxes across the guard cells plasma and tonoplast membranes. The double mutant displayed markedly reduced tonoplast vesicles, K^+^/H^+^ activity, and disruption in K^+^ accumulation in guard cells, which in turn may affect the guard cells osmoregulation capacity and stomatal movement (Andrés *et al.*, 2014). In addition, the *nhx1nhx2* mutant exhibited more acidic vacuoles and the disappearance of the highly dynamic remodeling of vacuolar structure associated with stomatal movements (Andrés *et al.*, 2014). Altogether, these data suggest that NHX1 and NHX2 are the main transporters mediating K^+^ uptake to the vacuole.

### pH homeostasis

Cellular pH homeostasis is one of the most important factors for cellular function. In plants cells, cytoplasmatic pH is regulated by the primary action of H^+^-pumps and metabolic process producing H^+^ or OH^−^. Cation/H^+^ antiporters constitute proton leak pathways allowing rapid cytoplasmatic pH adjustments (Rodríguez-Rosales *et al.*, 2009). Involvement of plant NHX antiporters in vacuolar pH regulation is best illustrated by studies of Japanese morning glory (*Ipomoea nil* or *Pharbitis nil*) flower petal coloration. During flower development, *Ipomea* petals begin to accumulate anthocyanins in vacuole, which are red at low pH but turn blue as pH increases. During the color transition, petal vacuolar pH shifts from ca. 6.5 to 7.5 and is accompanied by increased V-ATPase, V-PPase and InNHX1 expression and activity (Yamaguchi *et al.*, 2001; Yoshida *et al.*, 2009). The purple (*pr*) mutation of *Ipomea nil,* which abolishes the activity of *InNHX1*, partially hampers vacuole alkalinization and prevents the full color shift from red to blue in opening flowers (Yamaguchi *et al.*, 2001). The partial pH change and color in *pr* mutant has been suggested to rely on the activity of another abundantly expressed vacuolar NHX-type member, *InNHX2* (Ohnishi *et al.*, 2005).

### Vesicular trafficking

Eukaryotic cells synthesize, modify, and deliver molecular cargo in and between distinct cellular components through a complex and coordinated system of intracellular trafficking of cargo via vesicles. Vesicular trafficking depends on numerous molecular players and biochemical and biophysical factors. Among the principal factors, the vesicular luminal pH must be maintained within a narrow range that is unique to each specific intracellular compartment of the endomembrane system (Bassil *et al.*, 2012). *In vivo* pH measurements of intracellular compartments along the secretory pathway revealed a general gradual pH acidification with maturity, ranging from pH ~ 7.1 in the endoplasmatic reticulum to pH ~ 5.5 in the vacuole (Martiniere *et al.*, 2013). pH is critical not only for the compartmentalization of specific biochemical reactions but also for maintaining vesicular identity (through receptor association), sorting of newly synthesized or modified cargo, endocytosis, coat protein formation, energizing secondary transport system, as well as the degradation of molecules. The establishment of vesicular acidification is achieved by the action of V-ATPase and V-PPase. If vesicular pH was solely regulated by V-ATPase, the vesicular luminal pH could reach a pH below 3. Therefore, vesicular pH homeostasis is regulated by the active H^+^ transport mediated by H^+^-pump**s** and by luminal H^+^ leaks, thus establishing the optimal endosomal pH (Bassil *et al.*, 2012). In yeast, the function of ScNHX1 seems to be related to its involvement in protein sorting through endosomal pH regulation (Brett *et al.*, 2005; Rodríguez-Rosales *et al.*, 2009). Disruption of *ScNHX1* blocked the trafficking out from the Golgi/Prevacuolar compartment (Bowers *et al.*, 2000). The mutant also had an acidic cytoplasm and vacuole, as well as growth sensitivity to acidic media. In addition, protein processing and mis-sorting also occurred, because ~ 35% of the newly synthesized soluble vacuolar protein carboxypeptidase Y (CPY) was secreted to the apoplast (Bowers *et al.*, 2000; Bassil *et al.*, 2012).

In plants, the most direct evidence demonstrating a requirement for endosomal NHX antiporters in vesicular trafficking was generated using null mutants lacking both endosomal *AtNHX5* and *6* (Bassil *et al.*, 2011b; Reguera *et al.*, 2015). AtNHX5 and 6 reside in the Golgi, *trans*-Golgi network, and prevacuolar compartments (Reguera *et al.*, 2015). Plants lacking both *AtNHX5* and *6* displayed severely reduced growth (mainly due to reduced cell expansion), with smaller and fewer cells, and increased sensitivity to salt stress. In addition, trafficking of CPY was mis-sorted to the apoplast in the *nhx5nhx6* mutant, in a similar phenotype reported for *ScNHX1* disruption (Bowers *et al.*, 2000; Bassil *et al.*, 2011b, 2012). In a more recent report Reguera *et al.* (2015) fully characterized the *nhx5nhx6* mutant. The work showed that AtNHX5 and AtNHX6 are crucial for the maintenance of endomembrane luminal pH and supports the concept that proper vacuolar trafficking requires endomembrane pH homeostasis. In addition, transcriptional profile analyses of the Arabidopsis *nhx1* mutant revealed changes in the expression of a significant number of genes encoding proteins associated with intravesicular trafficking, trafficking to the nucleus, and Golgi processing (Sottosanto *et al.*, 2004). This indicates that, similar to what was reported for the yeast ortholog Nhx1p (Ali *et al.*, 2004), AtNHX1 also plays an important role in protein trafficking and targeting, probably via regulation of the intravesicular pH (Sottosanto *et al.*, 2004). Hamaji *et al.* (2009) reported that NHX1 is present in vesicles in the cytoplasm of salt-treated cells, suggesting that at least under salt stress, NHX1 may have a function in vesicular trafficking. Collectively, these reports indicate that endosomal NHX-type antiporters are critical regulators of endosomal trafficking likely by controlling the endosomal pH.

## Conclusion

Salt stress is a major constrain for agriculture worldwide. However, the development of salt-tolerant crops has been far too slow. There are many reasons delaying this progress, but the fact that salt-tolerance relies on the combined regulation of hundreds of genes, might be the main one. Plants have evolved remarkable mechanisms to regulate K^+^ and Na^+^ tissue and cellular homeostasis under salt stress. Many of these mechanisms relay on H^+^, K^+^ and Na^+^ transporters. Over the last years, several molecular studies have establish a strong involvement of SOS1, HKTs, and NHXs transporters in K^+^ and/or Na^+^ homeostasis and salt tolerance. Manipulation of some of these genes in model and crop plants yielded promising results in controlled conditions, but application in real agricultural conditions has been limited so far. The development of salt tolerant crops is unlikely to be successful until the development of new technical solutions allowing the fine-tuning regulation of multiple genes, preferentially in a tissue-specific manner. In addition, some of the ion transporters are also involved in key cellular processes, and overexpression of those genes can introduce excessive perturbations of related cellular and physiological processes, limiting the improvement of the salt stress response. Therefore, in order to provide critical insights for the development of salt-tolerant crop plants, future research efforts should be directed towards a better understanding of the molecular mechanisms (*e.g.* epigenetic modifications, transcription factors, post-translation modifications) underlying the regulation of those transporters.
